# Aliskiren Improves Ischemia- and Oxygen Glucose Deprivation-Induced Cardiac Injury through Activation of Autophagy and AMP-Activated Protein Kinase

**DOI:** 10.3389/fphar.2017.00819

**Published:** 2017-11-14

**Authors:** Ming-Hsien Chiang, Chan-Jung Liang, Chen-Wei Liu, Bo-Jhih Pan, Wen-Ping Chen, Yi-Fan Yang, I-Ta Lee, Jaw-Shiun Tsai, Chiang-Wen Lee, Yuh-Lien Chen

**Affiliations:** ^1^Department of Anatomy and Cell Biology, College of Medicine, National Taiwan University, Taipei, Taiwan; ^2^Lipid Science and Aging Research Center, Kaohsiung Medical University, Kaohsiung, Taiwan; ^3^Center for Lipid Biosciences, Kaohsiung Medical University Hospital, Kaohsiung, Taiwan; ^4^Institute of Pharmacology, College of Medicine, National Taiwan University, Taipei, Taiwan; ^5^Department of Internal Medicine, National Taiwan University Hospital, Taipei, Taiwan; ^6^School of Medicine, College of Medicine, China Medical University, Taichung, Taiwan; ^7^Department of Family Medicine, National Taiwan University Hospital, Taipei, Taiwan; ^8^Department of Nursing, Division of Basic Medical Sciences, and Chronic Diseases and Health Promotion Research Center, Chang Gung University of Science and Technology, Chia-Yi, Taiwan

**Keywords:** aliskiren, cardiac injury, oxygen glucose deprivation (OGD), apoptosis, autophagy

## Abstract

Aliskiren is a direct renin inhibitor that has been effective in anti-hypertension. We investigated whether aliskiren could improve the ischemia-induced cardiac injury and whether the autophagy was involved in this effect. A myocardial infarction (MI) model was created by the ligation of the left anterior coronary artery in C57J/BL6 mice. They were treated for 1, 3, 7, and 14 days with vehicle or aliskiren (25 mg/kg/day via subcutaneous injection). *In vivo*, the MI mice exhibited worse cardiac function by echocardiographic assessment and showed larger myocardial scarring by light microscopy, whereas aliskiren treatment reversed these effects, which were also associated with the changes in caspase-3 and Bcl-2 expression as well as in the number of apoptotic cells. Aliskiren increased autophagy, as demonstrated by LC3B-II expression and transmission electron microscopy. Furthermore, H9c2 cardiomyocytes were employed as an *in vitro* model to examine the effects of aliskiren on apoptosis and autophagy under oxygen glucose deprivation (OGD)-induced injury. Aliskiren significantly increased cell viability in a dose-dependent manner. The beneficial effects of aliskiren were associated with decreased apoptosis and mitochondrial membrane potential as well as increased autophagy via increased autophagosome formation. We also found that aliskiren-induced cardiomyocyte survival occurred via AMP-activated protein kinase (AMPK)-dependent autophagy. Taken together, these results indicated that aliskiren increased cardiomyocyte survival through increased autophagosomal formation and decreased apoptosis and necrosis via modulating AMPK expression. AMPK-dependent autophagy may represent a novel mechanism for aliskiren in ischemic cardiac disease therapy.

## Introduction

Cardiovascular disease (CVD) remains the leading cause of morbidity and mortality worldwide (Go et al., 2014). Ischemia is one of the major risk factors in CVD and causes extensive damages to myocardial tissues (Moe and Marín-García, 2016). Notably, apoptosis is the mainstay contributor to ischemia injury. Therefore, inhibiting myocardial cell death provides a rationale for the development of therapeutic agents against CVDs. However, autophagy, as another alternative mechanism, has caused an increasing awareness to determine cell fate (Tucka et al., 2012). Autophagy is an intracellular bulk degradation process in which cytosolic, long-lived proteins and damaged organelles are degraded and recycled (Matsui et al., 2008). Whether autophagy is protective in cardiomyocytes against stress is controversial because autophagy has been reported to have beneficial or detrimental effects on cardiomyocytes *in vitro* (Matsui et al., 2007). The role of autophagy remains unclear in CVD regarding whether the role of autophagy affects the cardiomyocytes from ischemia-induced death and drug treatment *in vivo*.

The progression of myocardial infarction (MI) was closely associated with the activation of the renin-angiotensin system (RAS) (Westermann et al., 2008). Increased RAS activity results in the development of cardiac hypertrophy, apoptosis, and left ventricular (LV) dilatation, all known to contribute to increased morbidity and mortality in patients with post-myocardial infarction and heart failure. Prevention of this RAS activity, which depresses cardiac function after MI, remains an important pharmacological target in treating patients with MI. Aliskiren is the only direct renin inhibitor that is clinically used as an oral drug to actively attenuate hypertension. Aliskiren is effective in attenuating the myocardial end-organ damage in hypertensive patients with LV hypertrophy (Solomon et al., 2009). In addition, aliskiren also improved ventricular function and cardiac remodeling in a mouse model of MI (Westermann et al., 2008). However, the mechanism of action remains unclear, as well as whether aliskiren modulates the post-MI environment. The intracellular signaling pathways by which ischemia-caused cell death occurs, are not well understood, but certain pathways have been proposed, including 5′ AMP-activated protein kinase (AMPK) (Qi and Young, 2015). Little is known about the effects of aliskiren on ischemia-induced cell death and the mechanisms of these effects. Obtaining a deeper understanding can provide important insights into the prevention of MI. In the present study, aliskiren was shown to increase survival by decreasing apoptosis and increasing autophagy, ultimately improving the beneficial effect of cardiomyocytes on overall LV function in MI-treated mice. Moreover, we showed that aliskiren upregulated AMPK phosphorylation in oxygen glucose deprivation (OGD)-treated cardiomyocytes, resulting in increased autophagy and attenuated apoptosis as well as subsequent increased cell viability.

## Materials and Methods

### *In Vivo* Mouse Model of Myocardial Infarction

All animal experimental procedures were performed in accordance with the “Guide for the Care and Use of Laboratory Animals” (NIH publication No. 86-23, revised 1985) and were approved by the Animal Care and Use Committee of National Taiwan University. Twelve-week-old male C57BL/6J mice (Laboratory Animal Center, National Taiwan University) were anesthetized and underwent surgery for the induction of MI by ligating the left anterior descending coronary artery (LAD). The LAD was ligated with 7-0 silk suture approximately 2 mm below the tip of the left atrial appendage. After 30 min of ligation, mice were randomly divided into two groups: mice + aliskiren (Norvatis, Basel, Switzerland) via subcutaneous injection (MI + ALI) and mice+PBS (MI). Aliskiren was given at 25 mg/kg once daily by subcutaneous injection after MI. We chose the dosage of aliskiren according to that used in a previous study (Westermann et al., 2008) and our pilot experiments (data not shown). Sham animals as the control underwent thoracotomy and incision of the pericardial sac, but not LAD ligation. The diagnosis of MI was confirmed by the presence of ST-segment elevation by electrocardiography.

### Physiological Assessments of Cardiac Function

The influence of the time course of MI and aliskiren on cardiac function was evaluated by echocardiographic assessment. Echocardiography was performed at 1, 3, 7, and 14 days after MI with a dedicated small-animal high-resolution ultrasound system (Prospect, S-Sharp, Taipei, Taiwan), equipped with a 40-MHz single-element transducer. M-mode tracings that were recorded from the long-axis view at the papillary muscle level of the left ventricle for the assessment of LV systolic function. LV fractional shortening (FS) % = (LVEDD-LVESD) /LVEDD x100, where LVESD and LVEDD represent LV end-systolic and end-diastolic diameters, respectively. All measurements were averaged for five consecutive cardiac cycles.

### Blood Pressure Measurements

Nine mice were randomly divided into three groups: mice + PBS and mice + aliskiren (25 mg/kg/day or 50 mg/kg/day) via subcutaneous injection for 14 days. The mice were anesthetized with pentobarbital (50 mg/kg, IP). Blood pressure (BP) was measured using the indirect tail-cuff method (BP2000, Visitech System, Apex, NC, United States).

### Specimen Collection and Morphometric Analysis

The mice were sacrificed by intraperitoneal administration of an overdose of pentobarbital at the indicated time points (1, 3, 7, and 14 days after MI). For RNA and protein extraction, cardiac tissue samples from the left ventricle of all groups were collected and preserved in liquid nitrogen. For morphometric analysis, the heart was dissected and transversely cut into three segments. The sections were immersion-fixed with 4% buffered paraformaldehyde and were routinely processed and paraffin embedded. The sections were stained with hematoxylin-eosin solution or with PicroSirius red staining (Picro-Sirius Red Stain Kit, Abcam). The LV wall thickness, LV wall area, infarct size and fibrotic area were analyzed using ImageJ.

### Real-Time PCR of Fresh Tissues

Total RNA was isolated from the powdered tissue according to the protocol supplied with TRIzol reagent (Invitrogen, Carlsbad, CA, United States) according to the manufacturer’s instructions.

cDNA was synthesized using the iScript cDNA synthesis kit (Bio-Rad Laboratories, Hercules, CA, United States). qPCR was performed using a Stratagene Mx3000p (Stratagene, La Jolla, CA, United States) and the SYBR-Green PCR kit (Applied Biosystems, Foster City, CA, United States). 18 s was used as an internal control to normalize each sample. The primers for COL1a and COL3a were used: COL1a, 5′-AAGAAGACATCCCTGAAGTCA-3′ and 5′-TTGT-GGCAGATACAGATCAAG-3′; COL3a, 5′-TTGGGATGCAGCCACCTTG-3′, 5′-CGCAAAGGACAGATCCTGAG-3′, respectively. Three independent experiments were performed in triplicate.

### Immunohistochemistry

For histochemical analysis of IL-1β, IL-6, TNF-α, LC3B and p-AMPK expression, 5-μm paraffin sections were incubated sequentially for 16 h at 4°C with antibodies against IL-1β (1:200, Proteintech), IL-6 (1:200, Abcam), TNF-α (1:200, GeneTex), LC3B (1:200, Epitomics), and p-AMPK (1:200, Abcam), and then for 90 min at room temperature with a 1:200 dilution of biotinylated goat anti-rabbit IgG antibody (Santa Cruz Biotechnology).

### Measurements of Angiotensin (Ang) II in Cardiac Tissues

To examine the effect of aliskiren on cardiac tissues, 20 mg of myocardial tissues from each group were homogenized in lysis buffer and then were centrifuged at 12,000 rpm for 15 min at 4°C. The supernatants were collected, and the levels of AngII were assayed using the Mouse ACE2 PicoKine ELISA kit (Boster, Pleasanton, CA, United States) according to the manufacturer’s instructions.

### Cell Culture and Oxygen Glucose Deprivation (OGD)-Induced Injury *in Vitro*

H9c2 cardiomyocytes were obtained from the American Type Culture Collection (ATCC CRL-1446TM, MD) and were grown in Dulbecco’s modified Eagle’s medium (DMEM, Gibco) supplemented with 10% fetal bovine serum, and 1% penicillin/streptomycin in a humidified atmosphere with 5% CO_2_ at 37°C.

After the cells had adhered, the cell culture medium was replaced with serum-free and glucose-free DMEM, and then the cells were placed in a 37°C incubator at 5% CO_2_, 94% N_2_, and 0.5% O_2_ for the indicated time as the OGD group (Karuppagounder et al., 2013). The environment mimicked the in vivo conditions of MI. Cells were treated for various periods in the presence or absence of aliskiren or other inhibitors. After the treatment, the cells were harvested and analyzed.

### Cell Viability Assay

H9c2 cells (4 × 10^4^ in 100 μL of medium) were seeded in 12-well plates. After the determined treatment, 100 μL of MTT stock solution (5 mg/mL, Thermo Fisher Scientific) was added to each well and was incubated for 30 min at 37°C. Next, 300 μL of dimethyl sulfoxide was added to each well to dissolve the formazan crystals. The plate was gently shaken for 10 min and was read at 550 nm on a spectrophotometer microplate reader. The optical density was used as the indicator of cell viability, and was normalized to that from the cells incubated in control medium, which were considered 100% viable.

### Flow Cytometric Analysis

Using annexin V and propidium iodide (PI) staining (Abcam), cell apoptosis was assessed by flow cytometry. After incubation for the indicated treatment, 10^5^ cells in 500 μL of binding buffer were mixed with 5 μL of annexin V-FITC, and then 5 μL of PI (50 μg/mL) was added to the cells, which were incubated at room temperature in the dark for 1 h. The stained cells were analyzed using FACSCaliber^TM^ flow cytometry (BD Biosciences, San Jose, CA, United States) and Cell-Quest software.

### Identification of Apoptosis by Terminal dUTP Nick End-Labeling (TUNEL) Staining

The TUNEL assay was performed on cells and tissues that were plated on glass slides using an *in situ* apoptosis detection kit according to the manufacturer’s instructions (In Situ Cell Death Detection Kit, Roche, CA, United States). The samples were incubated with the TUNEL reagent at 37°C for 1 h in a dark, humid chamber. The sections were counterstained with 4′, 6-diamidino-2-phenylindole (DAPI) to visualize all nuclei and were viewed under a fluorescence microscope. The number of TUNEL-positive nuclei was calculated from six non-overlapping regions of each tissue cross-section in a high-power field.

### Immunostaining for LC3B and p-AMPK

H9c2 cells were grown on cover slides in a 6-well plate. After treatment, cells were fixed in 4% paraformaldehyde in PBS, pH 7.4, for 15 min at 4°C. The cells were permeabilized with 1% Triton X-100 in PBS for 5 min, and then were incubated for 1 h at room temperature with LC3B antibody antibodies (1:50, Epitomics). After washing, the slides were incubated for 1 h at room temperature with fluorescein isothiocyanate-conjugated goat anti-rabbit IgG antibodies (1:100, Epitomics) and were viewed under a fluorescence microscope.

### Detection of Autophagosomes by Staining with Acridine Orange

Autophagosomes in the cells were detected by acridine orange staining as previously described (Traganos and Darzynkiewicz, 1994). Briefly, the cells were incubated with medium containing 1 μg/mL acridine orange (Invitrogen) for 15 min. The slides were observed by laser confocal microscopy (ZEISS, model LSM510) under Ex 488 nm with an Em filter setting in the band-pass at 520-560nm (green) and in the long-pass at 580 nm (red) to detect acidic vesicular compartment (red) and cytoplasma (green).

### Western Blotting

Western blot analyses were performed as described previously (Wang et al., 2012). 20 μg/mL of protein) was subjected to 10% SDS-PAGE and were then transferred onto PVDF membranes (Millipore, Billerica, MA, United States). The membranes were incubated overnight at 4°C with a 1:1000 dilution in TBST of primary antibodies against cleaved caspase-3 (1:1000, Cell Signaling), Bcl-2 (1:1000, Cell Signaling), LC3B I/II (1:1000, Cell Signaling), p-AKT (1:1000, Cell Signaling), total AKT (1:1000, Cell Signaling), p-AMPK (1: 1000, Abcam) or GAPDH (1:5000 dilution, Sigma), followed by incubation for 1 h at RT with horseradish peroxidase (HRP)-conjugated goat anti-rabbit/mouse IgG antibodies (1:5000, Cell Signaling) and bound antibody detected using Chemiluminescence Reagent Plus (NEN, Boston, MA, United States). The intensity of each band was quantified using a densitometer.

### Detection of the Mitochondrial Membrane Potential by JC-1 Staining

To investigate the effect of aliskiren on reducing OGD-induced cell death, changes in the mitochondrial membrane potential were measured by employing JC-1 (5,5′,6,6′-tetrachloro-1,1′,3,3′-tetraethylbenzimidazolylcarbocyanine iodide, Thermo Fisher Scientific). The monomeric form of the dye shows a green fluorescence (emission at 527 nm); however, within the mitochondrial matrix at high membrane potential, JC-1 forms aggregates that fluorescence red (emission at 590 nm). The cell suspension was incubated with JC-1 (5 μg/mL) dye for 15 min at 37°C, and green (FL-1) and red (FL-2) fluorescence was measured by fluorescence-activated cell sorting (FACS) and fluorescence microscopy.

### Transmission Electron Microscopy

The normal, borderline, and ischemic regions were excised and were fixed overnight at 4°C with 2% glutaraldehyde and 2% paraformaldehyde in PBS, pH 7.4, and were post-fixed for 1 h at RT with 1% OsO_4_. H9c2 cells were also collected by centrifugation and washed with PBS, followed by fixing with the same fixative, and post-fixing with 1% osmic acid. The samples were then dehydrated in graded ethanol, washed with propylene oxide, and embedded in epoxy resin. Ultrathin sections cut in a Reichert ultramicrotome were stained with lead citrate and uranyl acetate and were examined in a HITACHI H-7100 at 100 kV.

### Statistical Analysis

Values were presented as means ± SEM. The results of the animal study were analyzed by two-way ANOVA, followed by Tukey’s *post hoc* analysis. Other experiments were determined by one-way-ANOVA and the *post hoc* Dunnett test. The significance level was set at 0.05.

## Results

### Aliskiren Treatment Improves Cardiac Function and Morphology in MI Mice

**FIGURE 1:**
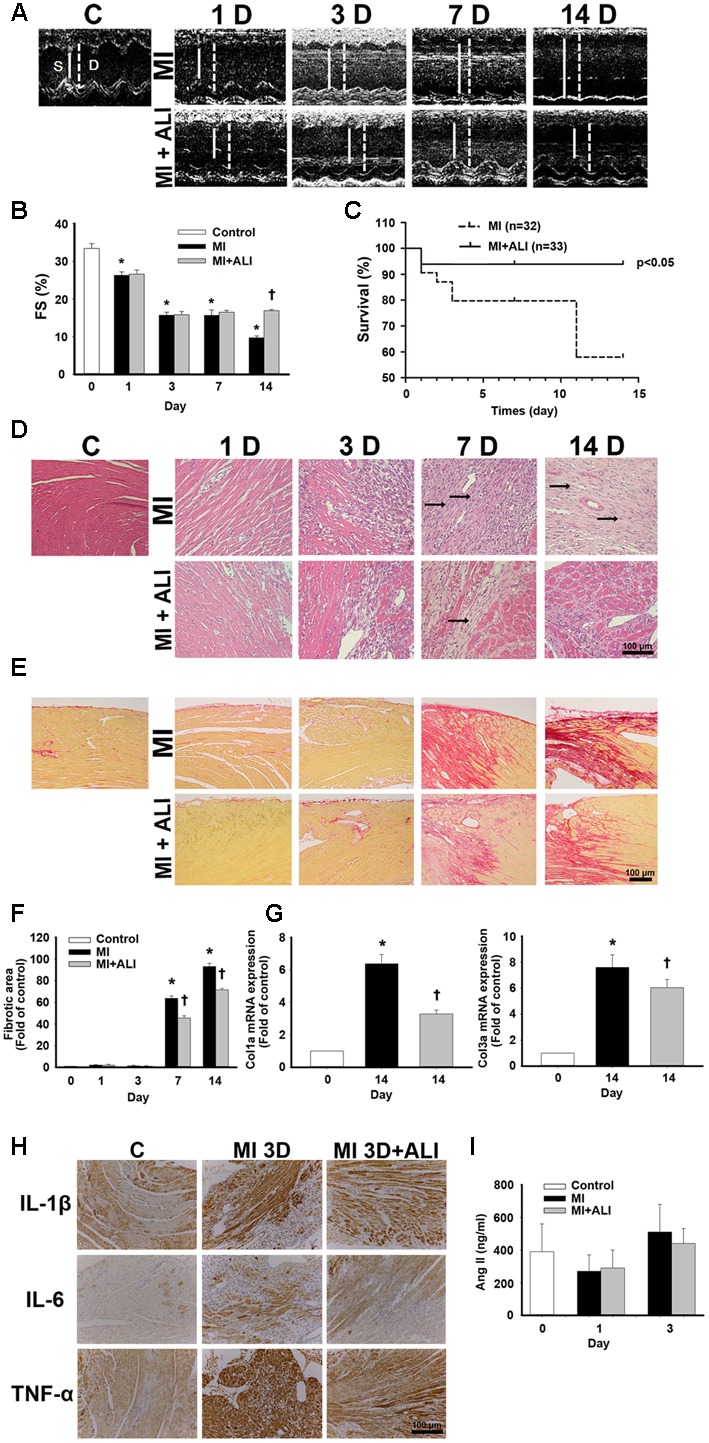
Aliskiren treatment improves cardiac function and morphology in MI mice. **(A)** Representative echocardiogram from PBS-treated mice (MI, *n* = 6) and aliskiren-treated mice (MI + ALI, *n* = 6) on 1, 3, 7, and 14 days after LAD ligation. The sham-operated mice (C, *n* = 17) showed a normal cardiograph. **(B)** Quantitative data of the fractional shortening (FS). **(C)** Survival rate at 14 days was following myocardial infarction (MI). Kaplan–Meier analysis revealed a trend of lower mortality in the aliskiren-treated mice (*n* = 33) than in the untreated mice (*n* = 32) (Mantel–Cox test, *p* = 0.0244.) **(D)** Representative histologic images of hematoxylin and eosin staining of MI mice (upper) and MI + ALI mice (lower). The arrows indicate the infarction area occupied with fibroblasts. Bar: 100 μm. **(E,F)** Representative pictures of PicroSirius Red staining **(E)** and statistical data **(F)** were presented in MI-treated mice and aliskiren-treated mice. Bar: 100 μm. **(G)** mRNA levels of collagen type1a and type 3a. **(H)** The representative pictures of the IL-1β, IL-6 and TNF-α expression in cardiac tissues at 3 days after MI and MI + ALI groups by immunohistochemistry. Bar: 100 μm. **(I)** Cardiac angiotensin II was determined in control and MI mice with and without aliskiren treatment at 1 and 3 days. All experiments were repeated at least three times, unless otherwise stated. The data are means ± SEM. ^∗^*P* < 0.05 compared with the control group. ^†^*P* < 0.05 compared with the MI group at the indicated times.

In the first set of experiments, we investigated whether aliskiren improved cardiac function in MI mice. **Figure 1A** shows representative M-mode images from sham-operated mice (C), PBS-treated MI mice (MI), and aliskiren-treated MI mice (MI+ALI) at 1, 3, 7, and 14 days. As shown in **Table 1**, LVEDD and LVESD in MI mice were significantly larger than those in control animals at the indicated times. Aliskiren treatment significantly attenuated LVEDD and LVESD in MI mice at 14 days. The fraction shortening (FS) in MI animals was markedly decreased compared with that in the control animals at the indicated time. By contrast, FS in the aliskiren-treated mice was significantly increased by 1.74 compared with that in PBS-treated mice at 14 days after MI (**Figure 1B**). Compared with MI mice, the aliskiren-treated mice showed an improved survival rate (*p* = 0.0244) (**Figure 1C**). We also examined whether aliskiren improved cardiac morphology in MI mice by hematoxylin and eosin staining. The area of cardiomyocytes was decreased in MI animals and was significantly increased with aliskiren treatment under higher magnification (**Figure 1D**). With MI progression, the myocardial infarct size was significantly smaller in the aliskiren-treated group compared with the MI group at 14 days. In addition, the LV wall thickness and LV wall area were dramatically decreased in a time-dependent manner; however, the reverse trend was observed with aliskiren treatment at 14 days (**Table 2**). Furthermore, the intense collagen deposition was present in the border zone of the heart after MI at 7 and 14 days by PicroSirius red staining (**Figure 1E**). Aliskiren treatment decreased the fibrotic area (**Figure 1F**). Moreover, MI significantly induced the expression of collagen type 1a mRNA and collagen type 3a mRNA, which are fibrotic markers, whereas aliskiren treatment markedly reduced their expression at 14 days by real-time PCR (**Figure 1G**). Furthermore, we examined whether aliskiren improved the inflammatory response in MI mice by immunohistochemistry. We found that the expression of IL-1β, IL-6 and TNF-α was strongly present in cardiac tissues of MI mice, while the expression was weak in aliskiren-treated mice at 3 days (**Figure 1H**). The levels of cardiac angiotensin II were not significantly changed among the three groups at 1 and 3 days (**Figure 1I**). Evaluation of the morphological changes in the left ventricle at 14 days showed less collagen deposition and increased numbers of cardiomyocytes in the ventricular wall in aliskiren-treated mice, confirming the positive functional imaging data seen in echocardiography.

**Table 1:** Myocardial infarction (MI) mice characteristics of the echocardiographic analysis.

Characteristics	LVESD (mm)	LVEDD (mm)
C (*n* = 17)	2.56 ± 0.07	4.02 ± 0.06
1D (*n* = 6)	3.19 ± 0.18^∗^	4.26 ± 0.22^∗^
1D + ALI (*n* = 6)	3.02 ± 0.15	4.04 ± 0.18
3D (*n* = 6)	3.49 ± 0.04^∗^	4.14 ± 0.05
3D + ALI (*n* = 6)	3.95 ± 0.34	4.57 ± 0.27
7D (*n* = 6)	4.07 ± 0.5^∗^	4.52 ± 0.43^∗^
7D + ALI (*n* = 6)	3.76 ± 0.18	4.55 ± 0.23
14D (*n* = 6)	4.81 ± 0.07^∗^	5.29 ± 0.14^∗^
14D + ALI (*n* = 6)	4.48 ± 0.47^†^	5.13 ± 0.48^†^

*Data are means ± SEMs. LVESD, left ventricular end-systolic diameter. LVEDD, left ventricular end-diastolic diameter. ^∗^*P* < 0.05 compared to the control group. ^†^*P* < 0.05 compared to the MI group at the indicated time.*

**Table 2:** Left ventricular (LV) classification of heart section.

Characteristics	Infarct	LV wall	LV wall
	size (%)	thickness (mm)	area (mm^2^)
C (*n* = 3)	0.35 ± 0.02	1.046 ± 0.105	10.12 ± 0.51
1D (*n* = 3)	1.06 ± 0.3	0.826 ± 0.098	6.56 ± 0.82^∗^
1D + ALI (*n* = 3)	0.89 ± 0.38	0.897 ± 0.104	7.48 ± 0.38
3D (*n* = 3)	0.67 ± 0.39	0.823 ± 0.093	7.34 ± 0.42^∗^
3D + ALI (*n* = 3)	0.46 ± 0.21	0.703 ± 0.077	7.20 ± 0.07
7D (*n* = 3)	40.55 ± 1.97^∗^	0.437 ± 0.027^∗^	5.25 ± 0.09^∗^
7D + ALI (*n* = 3)	25.48 ± 1.56^†^	0.502 ± 0.021	5.98 ± 0.12
14D (*n* = 3)	47.09 ± 1.66^∗^	0.375 ± 0.031^∗^	6.60 ± 0.03^∗^
14D + ALI (*n* = 3)	31.72 ± 2.00^†^	0.498 ± 0.042^†^	7.59 ± 0.40^†^

*Data are means ± SEMs. ^∗^*P* < 0.05 compared to the control group. ^†^*P* < 0.05 compared to the MI group at the indicated time.*

### Aliskiren Treatment Reduces Apoptosis and Increases Autophagy in MI Mice

**FIGURE 2:**
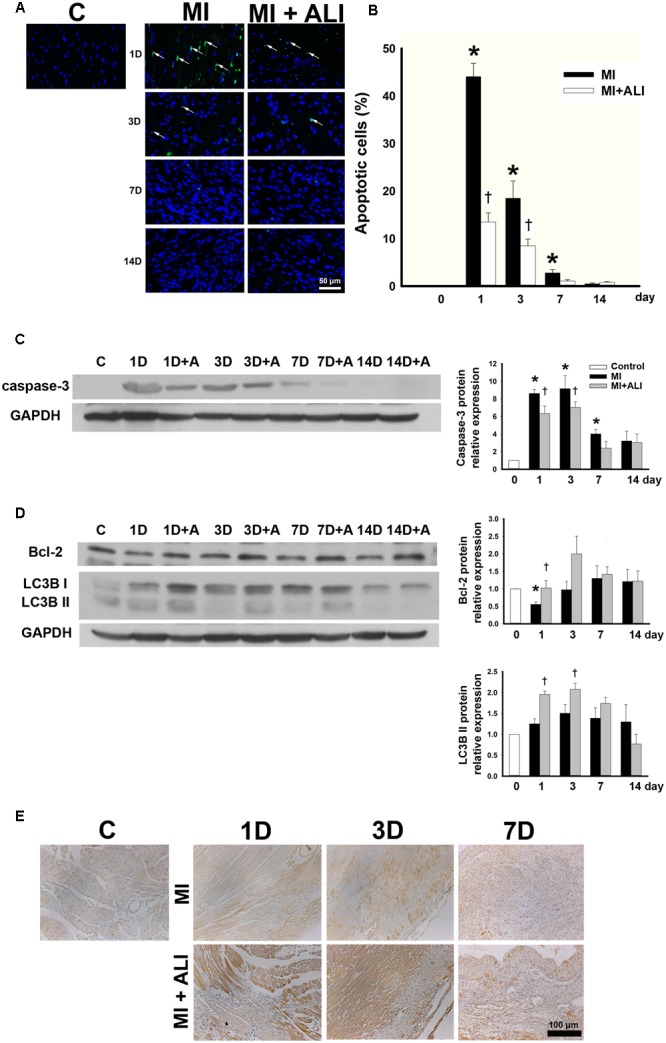
Aliskiren adminstration strongly decreases apoptosis and increases autophagy in MI mice. **(A)** TUNEL staining of the infarct zone of the control, MI and MI + ALI group at the indicated time (Blue: nuclei; green: apoptotic cell). Bar = 50 μm. **(B)** Apoptotic index as the percentage of TUNEL-positive nuclei compared with total nuclei. **(C,D)** The representative immunoblot and statistical analysis showed caspase-3 **(C)**, Bcl-2, and LC3B II **(D)** expression in cardiac samples. GAPDH was used as an internal control. **(E)** LC3B expression in cardiac tissues was stained by immunohistochemistry. Bar: 100 μm. Each of the experiments was repeated three times. The data are means ± SEM. ^∗^*P* < 0.05 compared with the control group. ^†^*P* < 0.05 compared with the MI group at the indicated times.

TUNEL-positive apoptotic cells were significantly present in the left ventricles of MI mice at 1 and 3 days after MI (**Figure 2A**). TUNEL-positive apoptotic cells were significantly decreased in the aliskiren-treated groups compared with those in the PBS-treated group at 1 and 3 days (**Figure 2B**). Caspase-3 expression was significantly increased in myocardial tissues at 1, 3, and 7 days after ischemia by Western blotting. Extensive suppression of caspase-3 was observed in aliskiren treatment at 1 and 3 days (**Figure 2C**). Moreover, the myocardial level of Bcl-2 was significantly increased in aliskiren-treated mice compared with MI mice after 1 day (**Figure 2D**). Therefore, all three assays (TUNEL assay, caspase-3, and Bcl-2 expression) demonstrated a significant increase in apoptosis at 1 day after ischemia, whereas reverses with aliskiren treatment. Furthermore, we examined the autophagy activity in myocardium by microtubule associated protein (LC3B) expression and specifically the ratio LC3B-II/GAPDH protein level by Western blotting. The LC3B-II ratio was slightly elevated in hearts following ischemia at 1D and 3D after MI. The LC3B-II expression in aliskiren treatment after ischemia was significantly higher than ischemia-alone treatment at 1 and 3 days (**Figure 2D**). Similar results were obtained by immunohistochemistry (**Figure 2E**). Stronger LC3B expression was closely associated with cardiomyocytes. These data indicated that significantly increased autophagy may contribute to the decreased MI injury following aliskiren treatment.

**FIGURE 3:**
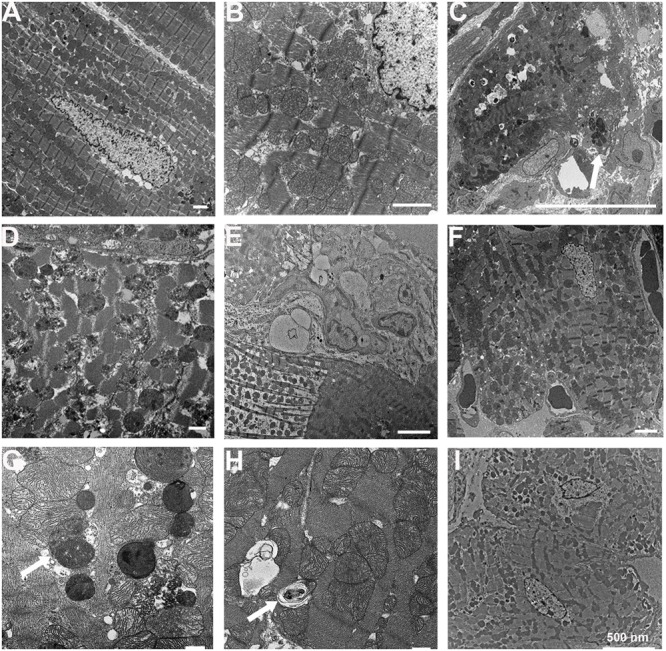
Aliskiren treatment improves cardiac ultrastructure and upregulates the autophagosomes in cardiomyocytes of MI mice by transmission electron microscopy. **(A)** Cardiomyocytes in the sham-operated mice. **(B)** The normal cardiomyocyte was shown at higher magnification. **(C)** The damaged cardiomyocytes in MI mice showed apoptotic bodies (arrow). **(D)** The damaged cardiomyocyte was shown at higher magnification. **(E)** Fibroblasts were shown in the infarcted and marginal myocardium at 14D following myocardial infarction. **(F)** Aliskiren improved the ultrastructure of cardiomyocytes. **(G)** Accumulation of autophagosomes in cardiomyocytes in aliskiren-treated mice. The arrow denotes the autophagosome. **(H)** Late autophagosomes contain concentric lamellar inclusions and cytoplasmatic contents (arrow). **(I)** Heart tissues from aliskiren treatment at MI after 14 days. Bar: 500 nm. Each of the experiments was repeated three times.

The cardiac tissues from sham-operated mice showed normal ultrastructure of cardiomyocytes by TEM (**Figure 3A**). They also showed normal morphology of mitochondria and sarcomeres at higher magnification (**Figure 3B**). The ultrastructures of the normal area from experimental groups were similar to those of the sham-operated group (data not shown). In the infarcted and marginal myocardium 1D following MI, there was evidence of damaged cardiomyocytes with large vacuoles and apoptotic bodies (**Figure 3C**). Cardiomyocytes in MI mice exhibited myofibrillar disorganization, segmental loss of myofibrils, mitochondrion rupture, and mitochondria with swelling, partial disappeared cristae, and dense structures (**Figure 3D**). The infarcted area at 3D following MI was similar to that in the 1D group (data not shown). In the infarcted and the marginal myocardium at 14D following MI, there was a marked increase in the number of fibroblasts (**Figure 3E**). Aliskiren improved the cardiac ultrastructure after MI (**Figure 3F**). Meanwhile, abundant autophagosomes with double membrane and containing cytoplasmic material such as mitochondria were observed in the aliskiren-treated group (**Figure 3G**). The late autophagosomes were a closed double-membrane bound vacuole and contained concentric lamellar inclusions and cytoplasmic contents (**Figure 3H**). Aliskiren treatment of MI after 14 days showed cardiomyocytes with normal ultrastructure and few fibroblasts (**Figure 3I**). These results suggested that aliskiren can activate the formation of autophagosomes.

### Aliskiren Treatment Increases Cell Viability in H9c2 Cells under OGD Challenge

**FIGURE 4:**
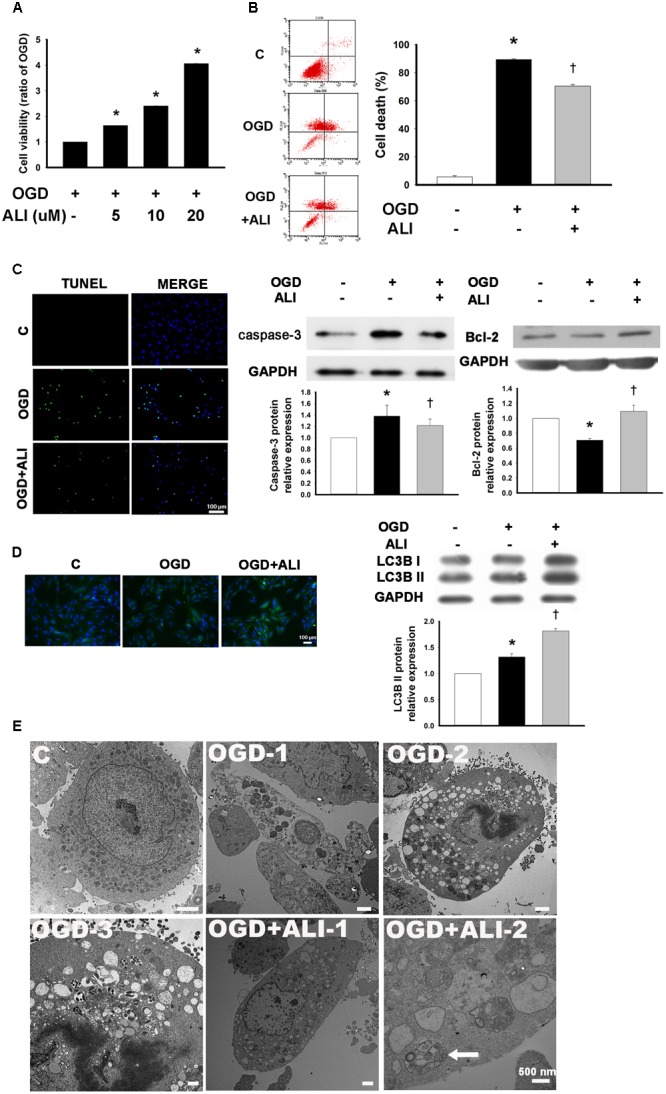
Aliskiren increases cell viability in oxygen glucose deprivation (OGD)-treated H9c2 cells via the upregulation of autophagosome formation. **(A)** Cardiomyocytes treated with 5, 10, or 20 μM of aliskiren were exposed to OGD for 16 h, and then cell viability was examined by the MTT assay. *^∗^P <* 0.05 compared with OGD. **(B,C)** Cardiomyocytes treated with 20 μM aliskiren were exposed to OGD for 16 h. Cell death was evaluated with Annexin V/PI staining by flow cytometer and TUNEL staining (Bar: 100 μm), and the protein expression of caspase-3 and Bcl-2 in OGD-treated H9c2 cells was assessed by Western blotting. **(D)** Effect of aliskiren on LC3B expression in OGD-treated H9c2 cells by immunocytochemistry (Bar: 100 μm) and Western blot. The ratio of LC3BII to GAPDH was quantified by using an imaging densitometer. **(E)** Ultrastructural changes of aliskiren on OGD-treated H9c2 cells by transmission electron microscopy. H9c2 cells appeared normal structure (C). Cell with OGD for 16 h showed necrosis (OGD-1) and apoptosis (OGD-2). Apoptotic cells displayed many vesicles and few autophagosomes in the cytoplasm (OGD-3). Many small vesicles were present in the cytoplasm (OGD + ALI-1). Abundant autophagsosomes (arrow) contained double membrane and cytoplasmic material such as mitochondria (OGD + ALI-2). Bar: 500 nm. Each of the experiments was repeated three times. The data are means ± SEM. GAPDH was used as an internal control. *^∗^P <* 0.05 compared with the control, ^†^*P <* 0.05 compared with OGD.

To determine the aliskiren working concentration, H9c2 cells were treated with 5–20 μM aliskiren for 16 h under normoxia, with no significant effect on cell viability by the MTT assay (data not shown). Aliskiren treatment rescued cell death in a dose-dependent manner in OGD-treated H9c2 cells (**Figure 4A**). Unless otherwise specified, 20 μM aliskiren was used in all subsequent experiments. The dead cells were significantly increased by OGD treatment, but the amount of dead cells in aliskiren-treated group was significantly attenuated compared with that in OGD-alone group by Annexin V/PI dual staining (**Figure 4B**). Similar results were obtained by TUNEL staining (**Figure 4C**). These changes were accompanied by an increase in caspase-3 expression in the OGD group, while the level of caspase-3 expression was significantly lower in the aliskiren group. We also found that aliskiren increased Bcl-2 expression in OGD-treated H9c2 cells (**Figure 4C**). These results demonstrate that aliskiren can reduce apoptosis via regulating caspase-3 and Bcl-2 expression. Furthermore, we investigated the effects of aliskiren on the autophagy. OGD-induced LC3B-II expression was significantly accelerated when the cells were treated with aliskiren by Western blotting. In addition, significant induction of autophagy by aliskiren in H9c2 cells under OGD was also confirmed by immunofluorescent staining (**Figure 4D**). These results suggested that aliskiren treatment enhanced the formation of the autophagy.

To confirm that aliskiren reduced apoptosis and increased autophagy compared with OGD-treated H9c2 cells, we compared the ultrastructural morphology among normal, OGD and OGD + aliskiren cells. TEM showed that H9c2 cells appeared to be normal with healthy nuclei (**Figure 4E**-C). Cells with OGD for 16 h showed necrosis (**Figure 4E**-OGD-1) and apoptosis (**Figure 4E**-OGD-2). Apoptotic cells displayed many vesicles and few autophagosomes in the cytoplasm (**Figure 4E**-OGD-3). Aliskiren improved the ultrastructure (**Figure 4E**-OGD + ALI-1). Meanwhile, abundant autophagsosomes with double membrane and containing cytoplasmic material such as mitochondria, were observed in the aliskiren groups (**Figure 4E**-OGD + ALI-2). These results suggest that aliskiren increases the formation of autophagosomes.

### Aliskiren Attenuates the OGD-Induced Decrease in the Mitochondrial Transmembrane Potential (MTP) in H9c2 Cells

**FIGURE 5:**
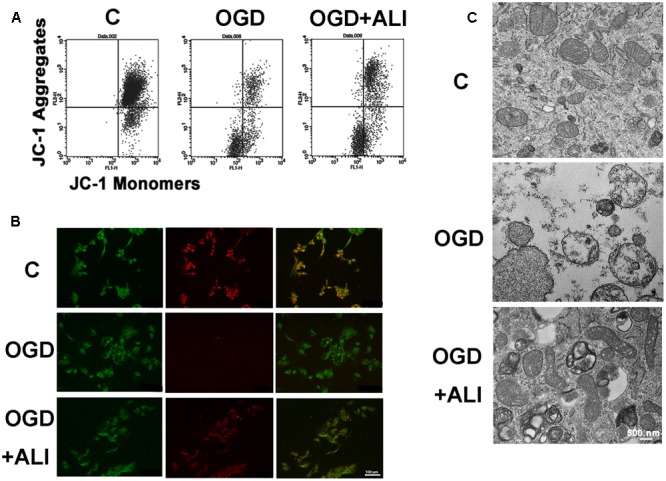
Aliskiren attenuates OGD-induced the reduction of mitochondrial transmembrane potential (MTP) and recovers mitochondrial intact in H9c2 cells. **(A)** Flow cytometric analysis of MTP by JC-1 staining. **(B)** Confocal microscopy of JC-1 staining from H9c2 cells with or without aliskiren under OGD for 16 h. Bar: 100 μm. **(C)** The effect of aliskiren on the ultrastructure of intact mitochondrial in OGD-treated H9c2 cells was examined by transmission electron microscopy. Bar: 500 nm. Each of the experiments was repeated three times.

To evaluate the protective effect of aliskiren on OGD-induced mitochondrial injury, if any, mitochondrial function was assessed using JC-1 staining to monitor MTP in cardiomyocytes. The number of cells with mitochondrial damage was examined by flow cytometry. Aliskiren increased the upper right fraction labeled by JC-1 as JC-1 red (intact fraction) and decreased the lower right fraction labeled by JC-1 as JC-1 green (damaged fraction) (**Figure 5A**). As shown in **Figure 5B**, normal cells displayed strong red fluorescence, revealing mitochondrial hyperpolarization. By contrast, cells with OGD treatment showed weak red fluorescence, demonstrating mitochondria depolarization and MTP reduction. Cells with aliskiren treatment displayed higher red fluorescence than OGD-alone treated cells. Consistent with the results from the MTP assay, OGD treatment induced mitochondria injury, whereas control and aliskiren-treated cardiomyocytes showed intact mitochondrial membrane as examined by transmission electron microscopy (**Figure 5C**). These results clearly indicate that aliskiren decreases mitochondrial depolarization, a key activity in cell death.

### Aliskiren Increases Cell Viability via the AMPK Pathway in H9c2 Cells during OGD-Induced Injury

**FIGURE 6:**
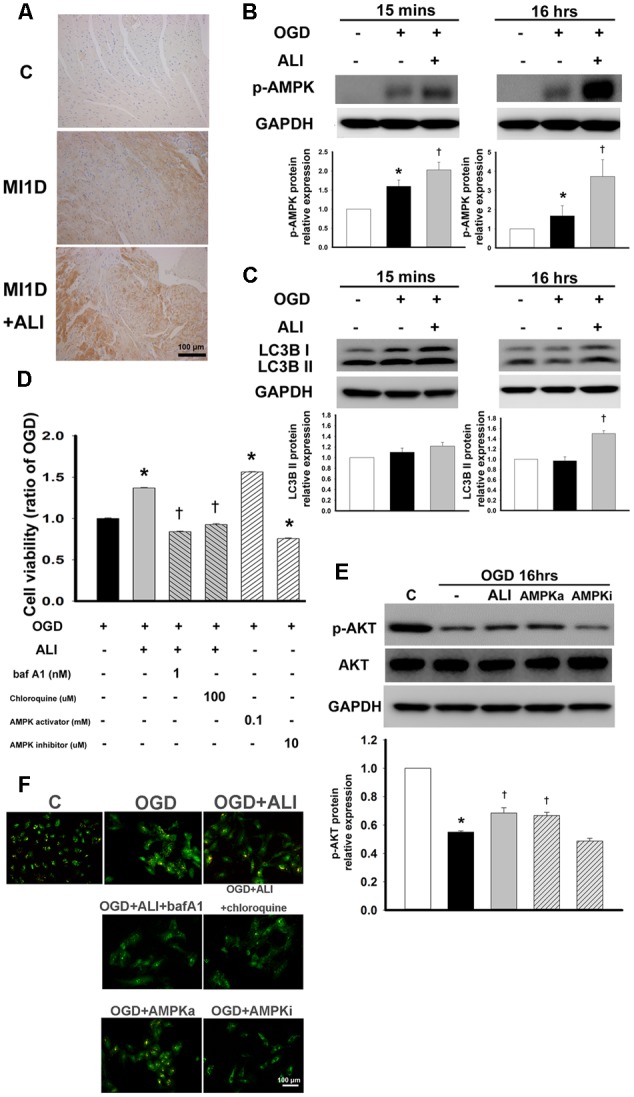
Aliskiren increases autophagy-induced cell viability by activation of the AMPK pathway in OGD-treated H9c2 cells. **(A)** Representative images of the effect of aliskiren on AMPK expression in cardiac tissues at 1 day after MI by immunohistochemistry. Bar: 100 μm. **(B,C)** H9c2 cells were treated with 20 μM aliskiren after OGD 15 min or 16 h, and then AMPK phophorylation and LC3BII expression were detected by Western blotting. **(D)** Cardiomyocytes co-treated with 20 μM aliskiren with or without bafilomycin A1 or chloroquine (autophagy inhibitors), or AICAR (an AMPK activator), or dorsomorphin (an AMPK inhibitor), were exposed to OGD for 16 h, and then cell viability was examined by the MTT assay. *^∗^P <* 0.05 compared with OGD. ^†^*P <* 0.05 compared with OGD + ALI. **(E)** Cardiomyocytes treated with 20 μM aliskiren, AICAR or dorsomorphin were exposed to OGD for 16 h, and then p-AKT and total AKT were examined by Western blotting. p-AKT was normalized to total AKT. **(F)** Autophagosomes were examined by acridine orange staining. Bar: 100 μm. Each of the experiments was repeated three times. The data are means ± SEM. GAPDH was used as the internal control. *^∗^P <* 0.05 compared with the control. ^†^*P <* 0.05 compared with OGD.

Next, we studied the underlying mechanism of autophagy-induced cell viability by aliskiren treatment. Therefore, we examined the level of AMPK activation in aliskiren-treated mice and H9c2 cells. As shown in **Figure 6A**, p-AMPK expression was mildly present in cardiomyocytes of the MI mice examined by immunohistochemical staining, while it was strongly present in aliskiren-treated mice. AMPK phosphorylation was increased in OGD-treated H9c2 cells. Aliskiren treatment was significantly increased at 15 min and 16 h (**Figure 6B**). Aliskiren-induced changes were accompanied by an increased level of LC3B-II expression at 16h (**Figure 6C**). To determine whether AMPK activity increased the cell viability in OGD-treated H9c2 cells, H9c2 cells were treated with an AMPK activator (AICAR) or an AMPK inhibitor (dorsomorphin) and then were subjected to the MTT assay and acridine orange staining (**Figure 6D** and 6F, respectively). The addition of the AMPK activator increased cell viability (**Figure 6D**), AKT phosphorylation (**Figure 6E**), and acridine orange staining (**Figure 6F**), whereas the AMPK inhibitor reduced these effects. To confirm the role of autophagy in cardioprotection induced by aliskiren, bafilomycin A1 or chloroquine (autophagy inhibitors) was applied in OGD-treated cardiomyocytes. Bafilomycin A1 and chloroquine potently reduced the cytoprotective effects of aliskiren (**Figure 6D**). In addition, cells treated with aliskiren, or with AMPK activator increased the formation of autophagosomes with OGD exposure as detected by acridine orange staining, whereas the AMPK inhibitor, bafilomycin A1 and chloroquine treatment decreased autophagosome formation (**Figure 6F**). The results suggest that aliskiren increases the cell viability via the upregulation of autophagy. These findings indicate that AMPK-AKT signaling was involved in the aliskiren-induced increase in cell viability and autophagy with OGD exposure.

## Discussion

The present study demonstrated that aliskiren treatment caused a significant improvement in FS compared with that in MI mice. The novel findings presented herein indicated that aliskiren reduced cell apoptosis and activated cytoprotective autophagy in ischemic hearts *in vivo* and in OGD-treated H9c2 cells *in vitro* by Western blot, light microscopy, and transmission electron microcopy. These results were closely associated with the increase in intact mitochondria in the cells. The AMPK-AKT pathway participated in aliskiren-induced cell viability. These findings favor the notion that aliskiren elicits protective effects on cardiac injury as an AMPK activator.

Aliskiren offers blocking tissue RAAS more effectively than angiotensin-converting enzyme inhibitors (ACEi) in the heart and does not reduce blood pressure more than ACEi or ARB (van Esch et al., 2010). The addition of aliskiren and losartan reduced urinary albumin excretion in patients with diabetic nephropathy (Parving et al., 2008). In addition, the addition of aliskiren to an ACEi in patients with chronic symptomatic heart failure resulted in lowering B-type natriuretic peptide in a 3-month study (McMurray et al., 2008). Aliskiren therapy reduced both end-systolic volume and end-diastolic volume in diabetic patients (Shah et al., 2012). By contrast, aliskiren did not improve LV remodeling with acute MI (Solomon et al., 2011). The ATMOSPERE study showed that there was not any different benefit between aliskiren and the ACEi enalapril therapy in patients with heart failure; however, the combination of both drugs resulted in more adverse events with no benefit despite the lowering of the plasma BNP levels (McMurray et al., 2016). Aliskiren does not influence the incidence of major cardiovascular events (Parving et al., 2012; Zhang et al., 2015). Based on the above findings, aliskiren has improved the surrogate markers for clinical outcomes in some, but not all, studies. A detailed understanding of the effect of aliskiren treatment and its related mechanisms was evaluated by multiple animal disease models. Aliskiren improved cardiac function and remodeling after MI (Westermann et al., 2008). Aliskiren reduced in the increase of the end systolic volume and hypertrophy index in mice post MI (Ramirez et al., 2014). Aliskiren also ameliorated isoproterenol-induced cardiac hypertrophy and apoptosis in rats (Bin-Dayel et al., 2016). The present study demonstrated that MI developed impaired cardiac contractility with severely reduced FS in a time-dependent manner by echocardiography. Treatment with aliskiren increased FS at 14 days after MI. Histologic analysis also demonstrated that the infarct area of all MI groups was increased compared with sham-operated mice in a time-dependent manner. Aliskiren treatment significantly reduced the infarct size. The results suggest that aliskiren treatment increased cardiac systolic and diastolic function and was found to be potent to decrease cardiac dilation, improving cardiac function and morphology.

Importantly, our pilot experiments showed different doses of aliskiren (25 mg/kg per day SC and 50 mg/kg per day SC) displayed no effects on blood pressure in sham-operated mice (**Table 3**). These results were consistent with previous study results showing that 10 and 50 mg/kg aliskiren showed no statistically significant lowering of the blood pressure (Westermann et al., 2008). Another previous study indicated that 30 mg/kg aliskiren did not significantly lower the blood pressure in SHR and WKY rats (Zhang et al., 2014). In addition, cardiac angiotensin II was not statistically significantly changed in control and MI mice with and without aliskiren treatment at 1 and 3 days (*P* > 0.05, **Figure 1I**). The results suggest that the improved effect of aliskiren on cardiac function and morphology was independent of blood pressure and the hormonal system.

**Table 3:** Blood pressure in sham-operated mice.

Characteristics	Control (*n* = 3)	Aliskiren 25 mg (*n* = 3)	Aliskiren 50 mg (*n* = 3)
Systolic blood pressure (mmHg)	124.3 ± 6.8	120.3 ± 7.8	125.3 ± 6.1
Diastolic blood pressure (mmHg)	73.3 ± 6.2	74.7 ± 8.2	73.7 ± 7.0

*Nine mice wee randomly divided into three groups: mice + PBS (*n* = 3), mice + aliskiren (25 mg/Kg/day, *n* = 3, SC) and mice + aliskiren (50 mg/Kg/day, *n* = 3, SC) for 14 days. Data are means ± SEMs.*

Myocardial infarction induced cardiac cell death and ischemic stress, triggering left ventricle remodeling and resulting in dilation, hypertrophy, and fibrosis (Konstantinidis et al., 2012). Cells die mainly by two modes of apoptosis and necrosis, and autophagy has been associated with cell death. Apoptosis contributes significantly to the loss of functioning cardiomyocytes during acute MI and subsequent remodeling events (Kitsis et al., 2007). Controlling cardiomyocyte loss after injury holds a therapeutic advantage because cardiomyocytes are terminally differentiated and have little potential for mitosis. In aliskiren-treated mice, the number of apoptotic cardiomyocytes and infarct size were reduced after MI (Westermann et al., 2008). Aliskiren also ameliorated cardiomyocytic apoptosis, attenuated the sympathetic nerve innervations and reduced the vulnerability of ventricular arrhythmias after MI (Jia et al., 2013). Aliskiren attenuated brain damage and decreased the levels of apoptosis in mice caused by acute cerebral ischemia (Miao et al., 2016). In the present study, we demonstrated that mice treated with aliskiren reduces the infarct size and incidence of cell apoptosis. Apoptotic cells were present in infarcted hearts (*in vivo*) and OGD-treated cardiomyocytes (*in vitro*) as detected by the TUNEL assay and TEM. We also showed that aliskiren significantly attenuates caspase-3 expression and increased Bcl-2 expression. Furthermore, a recent study highlighted that mitochondria are important in the regulation of cell death via both apoptosis and necrosis in addition to their key role in cell survival (Moe and Marín-García, 2016). The opening of permeability transition pores in the inner mitochondrial membrane with subsequent loss of ionic homeostasis and outer membrane rupture induce cell death (Javadov and Karmazyn, 2007). We demonstrated that aliskiren treatment preserved the MTPs by JC-1 flow cytometry, immunofluorescent staining, and TEM. The present study suggests that aliskiren may protect the heart from ischemic injury by promoting an intact mitochondrial membrane and Bcl-2 expression.

The present study demonstrated that aliskiren increased cell viability *in vivo* and *in vitro* studies under ischemia. Aliskiren inhibited apoptosis and necrosis, but it remained unclear whether autophagy was involved in aliskiren-triggered protection. The inhibition of autophagy exacerbated ischemia injury (Kanamori et al., 2011). Cardioprotective agents, such as adenosine and resveratrol have also been found to reduce damage dependent on autophagy induction (Kanamori et al., 2013). By contrast, aliskiren attenuated myocardial apoptosis via the decrease in autophagy in aged spontaneously hypertensive rats (Zhang et al., 2014). Aliskiren ameliorated pressure overload-induced heart hypertrophy and fibrosis in mice by suppressing autophagy (Weng et al., 2014). To date, a consensus has been reached that autophagy may be adaptive or maladaptive depending on the context in which it occurs and the extent of its stimulation (Nemchenko et al., 2011). The previous report revealed that autophagy as a repair mechanism is activated with low intensity stress (Vessoni et al., 2013). Modest levels of autophagy appear to increase cell survival through degrading and removing damaged mitochondria, therefore preventing the apoptosis. With increasing stress, apoptosis begins to occur. Under extreme stress, ATP depletion results in necrosis and neither autophagy nor apoptosis can progress (Nishida et al., 2008). Consistent with this, we found that autophagy may have been the initial response to mild stress, followed by the decrease in autophagy, as apoptosis and necrosis were increased in MI mice and OGD-treated cardiomyocytes. Furthermore, our data provided evidence that aliskiren leads to the activation of autophagy. The intriguing phenomenon was that aliskiren increased the amount of autophagy observed as autophagosomes by TEM and acridine orange-labeling vesicles. Aliskiren also elicited autophagy function, such as increased LC3B. In addition, autophagy inhibition with chloroquine or bafilomycin A1 resulted in the loss of aliskiren-induced protection including improved cell survival. These findings further support that autophagy displays beneficial effects in this study and aliskiren provides an advantage for cellular injury by enhancing autophagosomal formation.

To date, several cellular signaling pathways are speculated to activate autophagy (Qi and Young, 2015). AMPK activation turns on energy metabolism, gene transcription, cell mitosis and autophagy (Mihaylova and Shaw, 2011; Hardie et al., 2012). However, the beneficial outcomes of AMPK activation in cell survival remain controversial. Several studies have found that sustained AMPK activation under severe stress conditions may inhibit cell growth and accelerate cancer cell death (Chen et al., 2011; Kang et al., 2012). Others have found that AMPK activation has pro-survival potential (Narbonne and Roy, 2009; Jeon et al., 2012). One explanation is that, depending on the intensity of the stress, AMPK might coordinate with downstream kinases to rescue cells when facing minor or moderate stresses or to increase cell death when the rescue fails. Cardioprotective agents such as metformin and resveratrol have induced autophagy by facilitating AMPK activation (Kanamori et al., 2013). AMPK-dependent autophagy mediated the protective effect of the sonic hedgehog pathway on OGD-induced cardiomyocyte injury (Xiao et al., 2015). However, a dominant negative mutant of α2-AMPK in cardiomyocytes does not impair functional recovery (Folmes et al., 2009). In this study, strong activation of AMPK was induced by aliskiren, which was well-correlated with the induction of autophagy in cardiomyocytes under OGD. In addition to elevated AMPK expression with aliskiren during OGD, the AMPK activator increased cell viability, further indicating an important role of the AMPK mechanism in aliskiren-elicited cell viability.

The novel findings presented herein were that aliskiren increased autophagosomal formation and decreased apoptosis and necrosis in the cardiomyocytes of MI mice and in OGD-treated H9c2 cells. We also found that aliskiren treatment enhances autophagy via the activation of AMPK, which serves as a negative regulator against cell apoptosis under OGD. These findings provide new insight into the cardioprotective effects of aliskiren and can help develop new drugs for the prevention and therapy of cardiac diseases.

## Author Contributions

M-HC performed the experiments and prepared all figures; B-JP performed a part of the experiments; C-JL designed some experiments; C-WLiu, Y-FY, I-TL, J-ST, and C-WLee analyzed the data; W-PC provided the technique of the animal MI model; and Y-LC designed the research and wrote the manuscript. All authors reviewed and approved this manuscript.

## Conflict of Interest Statement

The authors declare that the research was conducted in the absence of any commercial or financial relationships that could be construed as a potential conflict of interest.
